# Cost-effectiveness of financial incentives to improve glycemic control in adults with diabetes: A pilot randomized controlled trial

**DOI:** 10.1371/journal.pone.0248762

**Published:** 2021-03-18

**Authors:** Leonard E. Egede, Rebekah J. Walker, Clara E. Dismuke-Greer, Sarah Pyzyk, Aprill Z. Dawson, Joni S. Williams, Jennifer A. Campbell

**Affiliations:** 1 Division of General Internal Medicine, Department of Medicine, Medical College of Wisconsin, Milwaukee, Wisconsin, United States of America; 2 Center for Advancing Population Science, Medical College of Wisconsin, Milwaukee, Wisconsin, United States of America; 3 Health Economics Resource Center (HERC), Palo Alto VA Health Care System, Palo Alto, California, United States of America; Universidad Miguel Hernandez de Elche, SPAIN

## Abstract

**Purpose:**

Determine the cost-effectiveness of three financial incentive structures in obtaining a 1% within group drop in HbA1c among adults with diabetes.

**Methods:**

60 African Americans with type 2 diabetes were randomized to one of three financial incentive structures and followed for 3-months. Group 1 (low frequency) received a single incentive for absolute HbA1c reduction, Group 2 (moderate frequency) received a two-part incentive for home testing of glucose and absolute HbA1c reduction and Group 3 (high frequency) received a multiple component incentive for home testing, attendance of weekly telephone education classes and absolute HbA1c reduction. The primary clinical outcome was HbA1c reduction within each arm at 3-months. Cost for each arm was calculated based on the cost of the intervention, cost of health care visits during the 3-month time frame, and cost of workdays missed from illness. Incremental cost effectiveness ratios (ICER) were calculated based on achieving a 1% within group drop in HbA1c and were bootstrapped with 1,000 replications.

**Results:**

The ICER to decrease HbA1c by 1% was $1,100 for all three arms, however, bootstrapped standard errors differed with Group 1 having twice the variation around the ICER coefficient as Groups 2 and 3. ICERs were statistically significant for Groups 2 and 3 (p<0.001) indicating they are cost effective interventions.

**Conclusions:**

Given ICERs of prior diabetes interventions range from $1,000-$4,000, a cost of $1,100 per 1% within group decrease in HbA1c is a promising intervention. Multi-component incentive structures seem to have the least variation in cost-effectiveness.

## Introduction

Thirty-four million adults, or 13% of the US population, have diabetes, with trends in prevalence continuing to increase [1]. The economic impact of diabetes is substantial, with a total estimated cost of $327 billion per year, an increase of 26% from estimates in 2012 [2]. Individuals diagnosed with diabetes account for 1 in 4 health care dollars spent at the national level, and individuals spend on average $16,750 per year [2]. These expenditures are 2.3 times higher than those without diabetes, and include costs attributed to lost productivity for those employed or reduced productivity for those not in the work force [2]. Lost productivity, when considered over the lifetime, suggests adults with diabetes have productivity adjusted life years 11–12 years shorter than individuals not diagnosed, impacting both quality of life and human capital at a societal level [3]. In addition, costs associated with diabetes on the Medicare program contribute an additional layer of societal cost as diabetes is one of the top three diseases accounting for personal health care spending in the US, and its prevalence in adults aged 65 and older continues to rise [2, 4].

Mounting evidence exists regarding the efficacy of financial incentives in initiating behavior change and improving health outcomes across conditions and behaviors, including smoking, weight loss, dietary behavior, vaccine completion, medication adherence and diabetes self-management [5–12]. Behavioral economics research proposes financial incentives can help individuals overcome the natural tendency to discount future consequences of poor health by providing an extrinsic reward in the short term [13]. For example, theory suggests that a vital factor of medication non-adherence is that patients do not perceive a clear cause-and-effect relationship between non-adherence and disease progression, so do not internalize consequences of non-adherence until it is too late [14, 15]. Studies suggest that incentive approaches improve adherence to treatment and improve some outcomes, but how incentives are structured, framed, and deployed can have substantial effect on success [10, 16–19]. For example, one project providing financial incentives to adult Medicaid beneficiaries found no significant differences in clinical outcomes or reduction in health cost, however, participants receiving incentives reported significantly higher adherence to healthy diet and significantly better physical health during the study compared to controls [20]. In addition, though individuals receiving financial incentives were more likely to conduct blood glucose self-monitoring and attend program meetings, they were not more likely to attend diabetic eye screening in a study conducted in the United Kingdom [12, 21].

An area with less investigation is how cost-effective financial incentives may be alone or in comparison to other types of interventions. This will be particularly useful to understand in the field of diabetes, given possible long-term cost savings by preventing complications resulting from uncontrolled blood sugar [1]. Systematic reviews conducted to understand the most cost-effective interventions for managing diabetes, its complications, and comorbidities found strong evidence that diabetes self-monitoring (DSME), compared with usual care, is very cost-effective ($5,047/Quality Adjusted Life Year, QALY) [22]. Multi-component interventions were also on average very cost-effective ($2,315/QALY) for individuals with diabetes compared with usual care [22]. Authors stressed the need for more cost effectiveness studies, underlining the fact that there may be additional cost-effective interventions which exist but have not been studied [22]. Given the limited number of studies on cost-effectiveness of financial incentive interventions, there is not enough evidence to draw strong conclusions. A validated microsimulation model study by Lee et al., revealed that incentives through food subsidy provided to Medicare and Medicaid participants over their lifetime was cost-effective at 5 years and beyond, with lifetime ICERs of $18,184/QALY (fruits and vegetable incentive) and $13,194/QALY (healthy food incentive) [23]. Despite the initial cost, financial incentives may be more acceptable to the wider society when aimed to benefit recipients beyond the incentive itself, such as an impact on long-term health [24].

Given the dearth of evidence in this area, the objective of this study was to use results from a small pilot study to determine the cost-effectiveness of three financial incentive structures provided to adults with diabetes in obtaining a 1% decrease in HbA1c.

## Materials and methods

### Study stetting, participants, and randomization

This study was a 3-group randomized trial with a follow-up period of three months. Study participants were recruited from general internal medicine, endocrinology, family medicine, and community primary care outpatient clinics at the Medical University of South Carolina (MUSC), an academic medical center located in Charleston, South Carolina. Institutional Review Board (IRB) approval was obtained for this study from the IRB at the Medical University of South Carolina. This study is registered at clinicaltrials.gov (identifier: NCT02722499).

#### Eligibility criteria

The inclusion criteria for the study included: 1) > = 21 years of age, 2) clinically diagnosed with type 2 diabetes and hemoglobin A1C > = 8.0% at time of screening, 3) self-identify as African American, 4) taking an oral medication or insulin for diabetes, 5) able to communicate in English, 6) access to a telephone and/or ethernet, and 7) willing to use the blood pressure and glucometer telemonitoring device during the course of the study. The exclusion criteria for the study included: 1) mental confusion suggesting significant dementia, 2) participation in another diabetes clinical trial, 3) alcohol or drug abuse/dependency, 4) active psychosis or acute mental disorder, 5) life expectancy <12 months.

A variety of recruitment methods were employed to identify and enroll interested study participants. This included systematic identification of African Americans with type 2 diabetes using billing records, and referrals from physicians, clinic staff, or patients themselves in response to recruitment flyers. Patients who expressed interest in participating were scheduled for a screening visit where a venous sample of blood was collected. Hemoglobin A1C (HbA1C) lab analysis was used to assess eligibility.

**Fig 1:**
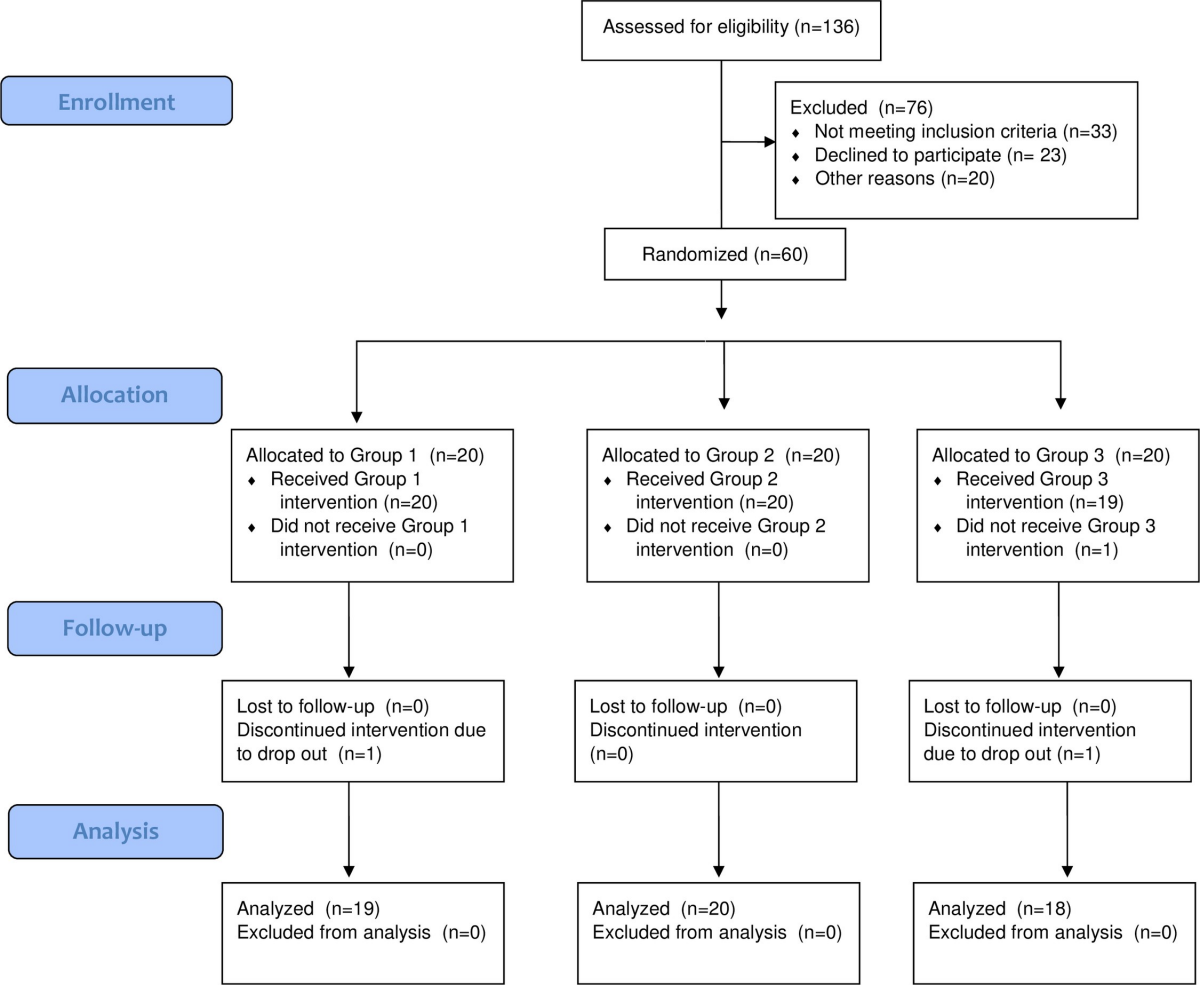
CONSORT flow diagram.

Once determined eligible, patients returned one week later to be consented and randomly assigned (1:1:1) to one of three study arms. A permuted block randomization method was used to assign participants to one of the three intervention groups (low frequency, moderate frequency, or high frequency). Block size was varied to minimize the blind being broken and randomization was stratified by baseline HbA1c levels (8–10% vs. >10%). Computer generated intervention assignment was obtained based on the pre-programmed randomization scheme, with all subjects who were randomized analyzed in accordance with CONSORT guidelines. Fig 1 provides the CONSORT diagram showing progress of participants through the trial.

### Intervention

Sixty (60) adult African Americans with type 2 diabetes were randomized to one of three study arms between March 1, 2016 through July 31, 2016. The consort diagram for study procedures can be found in Fig 1. All study participants were provided 1) telephone-delivered diabetes education and skills training for 12 weeks with a trained nurse educator, and 2) one of three structured financial incentives.

#### Telephone-delivered diabetes education and skills training

Registered nurses completed weekly telephone calls with participants for 12-weeks. Sessions lasted approximately 30 minutes and included discussions on diabetes knowledge and information, patient activation, patient empowerment, and behavioral skills training. Participants were provided with test strips and were asked to upload at least one blood pressure and one glucose reading each day. The home tele-monitoring device then loaded readings into a secure server that was accessed by the nurse health educator in real time.

#### Financial incentives

Each participant received one of three structured incentives, receiving up to $300 based on conditions of the group to which they were assigned:

- **Low Frequency Financial Incentive (Group 1)** received a single incentive at 3-months for absolute percentage drop in HbA1c from baseline to 3-month follow-up, up to $300.
- **Moderate Frequency Financial Incentive (Group 2)** received a two-part incentive for uploading glucose measurements through home testing of glucose and absolute percentage drop in HbA1c from baseline to 3-month follow-up, up to $300.
- **High Frequency Financial Incentive (Group 3)** received a multiple component incentive for uploading glucose measurements using home testing, attending weekly phone educational sessions, and absolute percentage drop in HbA1c from baseline to 3-month follow-up, up to $300.

### Measurement of clinical outcome

The primary clinical outcome of interest was HbA1c reduction within each arm at 3-months post randomization. HbA1c was measured at baseline and at 3-months post randomization and the difference for each individual was calculated. Primary outcome analyses investigated within group change in HbA1c from baseline to 3-months in each treatment arm. Secondary outcome analyses investigated within group change in blood pressure, quality of life, diabetes knowledge and self-care behaviors from baseline to 3-months in each treatment arm. Given the clinical benefit of 1% drop in HbA1c in reducing complications, this level was chosen as the clinically meaningful outcome.

### Measurement of cost

We followed methodology used by Pyne et al. for calculation of cost-effectiveness, substituting HbA1c for Quality Adjusted Life Years to measure effectiveness [25]. Economic outcomes included cost of the intervention, cost of health services, and cost of missed workdays. All costs were adjusted using the US Department of Labor Inflation Calculator to 2018 dollars [26].

### Cost for the intervention

1) *Personnel*. Calculated based on staffing of a registered nurse who provided home monitoring and telephone-based education ($65,000 salary plus 20% fringe) and was applied equally to the three treatment groups at $26,000 per arm.

2) *Incentive*. Each participant could receive up to $300, so incentive costs totaled $6,000 per arm for 20 participants in each treatment group. Though the total amount each individual received was based on meeting conditions of the incentive group to which they were randomized, and may be less than $300, we used an incentive cost of $300 for each participant in the analysis to ensure the most conservative cost-effectiveness estimates possible in regard to the incentive costs.

3) *Technology*. The home telemonitoring device, test strips, lancing device and web system had a mean cost of $105 dollars per patient in 2012 costs when devices were purchased. Using the US Department of Labor Inflation Calculator, this cost would be $117 in 2018-dollar value. For each treatment group the cost of 20 patients was calculated ($2,340).

4) *Space*. Cost for institutional overhead was calculated based on renting a 150 sq. ft. office at the rate of $1.50 a sq. ft. for 3 months ($675) and was applied equally across the three treatment groups. This cost was based on 2012 dollars, so it was adjusted for inflation using the U.S. Department of Labor Inflation Calculator ($250 per arm in 2018-dollar value).

Summing the personnel cost, incentives, technology, and space resulted in $34,590 per treatment group. Distributing these costs per patient, the cost was $1,729.50 per patient. Connectivity costs were not included in the measures due to these costs being a standard, fixed costs that were not specific to, nor influenced by the intervention.

#### Cost for health services

The cost estimates for health services utilization per participant were estimated using the number of each category of health services utilization per participant at 3 months follow-up multiplied by the 2015 Medical Expenditure Panel Survey (MEPS) mean expenditure per service in that category (Emergency Department Visits, Physician Visits, Outpatient Hospital Visits, and Inpatient Visits) [27]. The U.S. Department of Labor Inflation Calculator was then used to convert the 2015 expenditures to 2018-dollar values [26].

#### Cost for missed workdays

Cost for missed workdays was estimated first by using interval regression to estimate a predicted value of income for each participant since income was reported in intervals. The interval regression included age, gender, race and education as predictors. The predicted value of income was then divided by 260 days to attain an estimated per day salary value. This value was multiplied by the number of missed workdays each participant reported at 3-month follow up. As with health services cost, cost for missed workdays was adjusted for inflation using the U.S. Department of Labor Inflation Calculator to 2018-dollar values [26].

### Statistical analysis

Participants randomized per protocol were used for the analysis, with significance considered at p<0.05. Descriptive statistics were calculated overall and stratified by intervention group (low, moderate, or high frequency) for demographic factors and the clinical outcome at baseline and at the 3-month follow-up. Missing data was presumed to be missing at random and multiple imputation was used to account for missingness. Effectiveness was defined as 1% HbA1c within group change from baseline to 3-months post randomization. For calculation of total cost per patient, the cost of intervention, healthcare costs, and missed workdays were summed. Incremental cost effectiveness ratios (ICER) were compared to a specified monetary threshold, which represents the maximum amount that the decision-maker is willing to pay for health effects (maximum acceptable ceiling ratio). The sample was bootstrapped with replacement of 1,000 replications to provide confidence intervals. The intervention was deemed cost-effective if the ICER fell below this threshold and not cost-effective otherwise. All analyses were performed using STATA version 15.0 (StataCorp, College Station, TX).

## Results

Table 1 provides demographics of the sample overall and by intervention group. Mean age was 57.4 years, on average participants had been diagnosed with diabetes for 16.6 years, and 70.5% were female. There were no significant differences in demographics or HbA1c at baseline by intervention arm.

**Table 1:** Sample demographics for study overall and by intervention group.

	Overall Sample	Group 1 (Low Frequency)	Group 2 (Moderate Frequency)	Group 3 (High Frequency)	p-value
Age	57.4 (11.4)	58.1 (14.1)	57.5 (8.8)	58.2 (9.6)	0.11
Duration of Diabetes	16.6 (10.8)	18.6 (13.4)	17.7 (9.2)	17.2 (11.4)	0.30
Years of Education	13.1 (2.8)	12.8 (1.7)	12.3 (1.8)	14.9 (2.6)	0.11
Gender					
Male	29.5%	25%	37.5%	37.5%	0.76
Female	70.5%	36.6%	29.3%	34.2%	
Marital Status					
Married	29.6%	37.5%	12.5%	50%	0.13
Not Married	70.4%	31.7%	39%	29.3%	
Income					
Less than $20,000	51.1%	40%	40%	20%	0.33
$20,000-$49,999	30.7%	27.3%	27.3%	45.5%	
$50,000 and more	18.2%	30%	20%	50%	
HbA1c, mean (95% CI)		10.0 (8.9, 11.0)	9.9 (9.2, 10.6)	10.4 (9.3, 11.5)	

Table 2 provides the mean drop in HbA1c, and p-value for within group change from baseline to 3-months follow-up for each treatment group. Group 1 (Low Frequency) participants dropped on average 1.25% HbA1c, which was significantly different from baseline (p = 0.002). Group 2 (Moderate Frequency) participants dropped on average 1.73% HbA1c, which was significantly different from baseline (p<0.001). Group 3 (High Frequency) participants dropped on average 1.74% HbA1c, which was significantly different from baseline (p<0.001).

**Table 2:** Clinical outcome (3-month mean drop in HbA1c) by incentive group.

Group	Mean Drop in HbA1c	p-value
1 (Low Frequency)	- 1.25%	0.002
2 (Moderate Frequency)	- 1.73%	<0.001
3 (High Frequency)	- 1.74%	<0.001

Table 3 provides the costs by intervention. Intervention costs for each group was $1,729.50. Mean per person costs for each group were calculated at $3,765 for Group 1, $4,649 for Group 2, and $3,315 for Group 3.

**Table 3:** Cost by incentive group.

	Group 1—Low Frequency (mean ± sd)	Group 2 –Moderate Frequency (mean ± sd)	Group 3 –High Frequency (mean ± sd)
Costs			
Intervention	$1,729.50	$1,729.50	$1,729.50
Primary Care	$348.95 ± $297.56	$282.39 ± $369.85	$243.10 ± $188.35
Other Health Care	$1,352.84 ± $2,511.60	$1,020.00 ± $2,268.14	$780.30 ± $1,043.50
ED Visits	$301.58 ± $1,105.16	$795.83 ± $3,146.13	$334.25 ± $1,494.81
Workdays Missed	$32.70 ± $131.24	$821.96 ± $3,416.88	$228.30 ± $476.15
**Total**	**$3,765.57 ± $2,765.57**	**$4,649.68 ± $5,393.52**	**$3,315.45 ± $2,014.43**

Table 4 provides the ICERs for each treatment group and p-value for cost-effectiveness of the group in obtaining a 1% within group decrease in HbA1c. The ICER for all three treatment groups was $1,100, however Group 1 had twice the variation as Groups 2 and 3 and was not significant at a p<0.05 cut point.

**Table 4:** Bootstrap analysis of Incremental Cost Effectiveness Ratios (ICERs).

Intervention Group	ICER Coefficient	Standard Error	95% CI	p-value
1 (Low Frequency)	-1,100.24	644.02	(-2362.50,162.02)	0.09
2 (Moderate Frequency)	-1,100.24	314.54	(-1716.72, -483.76)	<0.001
3 (High Frequency)	-1,100.24	228.98	(-1549.03, -651.45)	<0.001

## Discussion

In this study, we found incremental cost effectiveness ratios (ICERs) for three structured financial incentives in obtaining a 1% within group decrease in HbA1c to be $1,100. Given ICERs of prior diabetes interventions range from $1,000-$4,000 [28, 29], a cost of $1,100 per 1% within group decrease in HbA1c is a promising intervention. Though the cost of financial incentives may be larger than many behavioral interventions, results suggest costs incurred through financial incentive systems could have long term cost savings in health for diabetes populations. Groups 2 (moderate frequency) and 3 (high frequency) resulted in larger overall drops in HbA1c, had less overall variation in cost effectiveness, and had ICERs significant at a p<0.05 level, indicating more frequent and smaller incentives may be more effective in initiating behavior change than larger but less frequent incentives.

This study provides important preliminary evidence that financial incentives are a potentially cost-effective intervention for individuals with diabetes who are not meeting glycemic targets, and that multiple component incentive structures should be investigated further. Results of this study align with findings from a recent systematic review on cost-effectiveness of interventions to manage diabetes, that multiple component interventions focused on managing diabetes and risk factors for developing complications is very cost effective [22]. Though the evidence that interventions to manage diabetes are cost-effective grew between a review conducted in 2010 and the most recent review, no financial incentive interventions were included in either systematic review [22, 30]. The finding that multicomponent interventions were very cost effective compared to usual care, combined with these findings that multi-component financial incentive structures had less variation in cost-effectiveness and resulted in larger drops in HbA1c, suggest that adding small financial incentives that match to the different components of previously tested interventions may offer an additional factor to initiate behavior change.

In addition to providing unique information on the cost-effectiveness of financial incentives in improving glycemic control, this study offers recommendations on next steps for investigation regarding financial incentives and diabetes self-management. First, as a pilot study, follow-up was limited to 3-months after randomization. A number of studies have suggested that long-term maintenance of behavior initiated by financial incentives needs investigation [6, 7, 10]. Within the context of diabetes self-management, this should incorporate collection of multiple behaviors over time to investigate if specific behaviors erode faster or change less following incentivization. In addition, one controversy regarding financial incentives is that patients may be less inclined to follow future clinical recommendations when incentives are removed. An ongoing study is currently investigating financial incentives and nurse coaching with a longer follow-up period of 12-months and incorporation of sustainability 6-months after the financial incentive is withdrawn (18-months after randomization) [31]. This study will help investigate whether long-term maintenance and sustainability of intervention effect exists for structured incentives in adults with diabetes, however additional studies are needed assessing a range of settings and different populations. Secondly, while the direction and magnitude of cost-effectiveness was similar across the various incentive structures, variability in the ICER and magnitude of the change in HbA1c differed. Future investigation is needed to understand if specific behaviors are best incentivized financially. Additionally, research should incorporate collection of clinical outcomes, such as HbA1c, as opposed to concentration only on behavior change. This information will provide a more comprehensive understanding of how to incentivize not only short-term behavior change, but long-term clinical outcomes and prevention of complications. Third, research suggests that even small financial incentives can influence health behaviors [17], which aligns with the findings of this study that smaller, more frequent incentives resulted in less variability in the cost-effectiveness of the intervention. This has implications for design of future financial incentive studies and suggests that providing smaller amounts but linking incentives to more frequent behaviors could result in a more cost-effective overall intervention. Though financial incentives are relatively expensive interventions, if they are effective at preventing expensive long-term complications, interest by health systems and policy makers may be higher. While some individuals find financial incentives unacceptable to promote healthy behavior, research suggests the public considers them more acceptable when they are seen as benefiting the wider society or are aimed to benefit recipients beyond the incentive itself [24]. Benefits related to decreased costs for health systems may offer a more societal perspective on the utility of incentives. Finally, studies are needed to investigate heterogeneity of effect, and determine if specific groups respond more positively or negatively to financial incentives. In this study there were more individuals with higher education and higher income in the high frequency incentive group, though differences between groups were not statistically significant. Differences by racial/ethnic groups will be evaluated in the ongoing study noted above [31], however, investigation into differential effects by age, gender, and socioeconomic status may help target specific types of incentive programs to groups in which they are most cost effective.

In conclusion, this pilot study using three financial incentive structures, estimated overall it cost $1,100 to obtain a 1% within group decrease in HbA1c. Costs incurred through multi-component financial incentive systems may have long term cost savings in health for diabetes populations and result in less variability across populations not currently meeting glycemic targets. Overall, this is a promising intervention, and highlights the need for cost-effectiveness analyses on new efforts to manage diabetes and minimize diabetes related complications.

## Supporting information

S1 Checklist CONSORT 2010 checklist of information to include when reporting a randomised trial*. (DOC)

S1 Protocol (PDF)
