# Supplementary material for: Cost-effectiveness of financial incentives to improve glycemic control in adults with diabetes: A pilot randomized controlled trial
Source: PLoS One. 2021 Mar 18;16(3):e0248762. doi: 10.1371/journal.pone.0248762 (PMC7971847; doi:10.1371/journal.pone.0248762)
Supplement: S1 Protocol — (PDF) [file pone.0248762.s002.pdf]

Financial Incentives Augmented Telephone Education and Skills Trial in African Americans with Diabetes (FITEST)
Principal Investigator: Leonard E. Egede, MD, MS

A: SPECIFIC AIMS

Diabetes Mellitus affects approximately 24 million adults in the United States (NIH 2008). African Americans (AA) with Type 2 diabetes (T2DM) have higher prevalence of diabetes, poorer metabolic control (i.e. poorer blood glucose, blood pressure, and lipid control), and greater risk for complications and death compared to White Americans (NIH 2008). Behavioral economics research has identified several decision-making patterns that contribute to poor adherence to medical regimens. Delay discounting represents the extent to which consequences or outcomes decrease in their effectiveness to control behavior the longer the consequences or outcomes are delayed (Reynolds 2006). Therefore, adults with diabetes who prefer smaller, immediate benefits (e.g. eating preferred foods or not being inconvenienced by exercising or taking medications) over larger, delayed benefits (e.g. better metabolic control, reduced risk of complications, or reduced risk of dying), are less likely to make healthy lifestyle decisions. Using financial incentives to promote medical adherence and healthy behaviors is consistent with behavioral economic theory and findings (Sutherland 2008). We propose a pilot randomized controlled study to test the efficacy of financial incentives augmented telephone-delivered diabetes education and skills training intervention compared to usual care in improving metabolic control in AAs with poorly controlled T2DM. The long-term goal of the project is to identify effective strategies to improve metabolic control and hence reduce diabetes complication and mortality rates in AAs with T2DM.

Primary Objective

To test the efficacy of three financial incentive structures in combination with technology intensified diabetes education and skills training intervention on HbA1c levels in AAs with T2DM.

Secondary Objectives

1. To test the efficacy of three financial incentive structures in combination with technology intensified diabetes education and skills training intervention on blood pressure and quality of life in AAs with T2DM.

2. To test the efficacy of three financial incentive structures in combination with technology intensified diabetes education and skills training intervention on diabetes knowledge and self-care behaviors (medication adherence, diet, exercise, and self-monitoring of blood glucose).

RESEARCH DESIGN AND METHODS

Study Overview. The overarching aim of this proposal is test the efficacy of three financial incentive structures in combination with technology intensified diabetes education and skills training intervention on blood pressure and quality of life in AAs with T2DM. 60 AAs with T2DM will be randomized to three groups with varying frequency of financial incentives: 1) High Frequency: financial incentives for weekly uploads plus average glucose, incentives for weekly attendance to educational sessions, and incentives at the end of the study for meeting HbA1c goals 2) Moderate Frequency: financial incentives for weekly uploads plus average glucose, and incentives at the end of the study for meeting HbA1c goals, and 3) Low Frequency: financial incentives at the end of the study for meeting HbA1c goals.

Study Population & Recruitment Plan

<u>Study clinics</u>: The study sites for this study are MUSC general medicine, endocrine, family medicine, and community primary care clinics. The investigative team has access to patients in these clinics and letters of support from key physician collaborators in each of these clinics are attached.
<u>Recruitment Strategy</u>: We will use two complementary approaches to identify eligible study subjects. The first method will consist of systematic identification of AA patients with T2DM. After obtaining IRB approval for a partial waiver of HIPAA, we will use clinic-billing records over the previous 12-month period to identify AA subjects with ICD-9 codes consistent with a diagnosis of T2DM. The physicians of eligible patients will be notified of their patients' potential eligibility and asked permission to enroll their patients in this study. The

second method will consist of referrals from physicians, other clinic staff such as nurses, or patients themselves in response to recruitment flyers for the study.
Participant Payment: Participants will receive $25 each for completion of baseline and 3 month assessments for a total payment of $50.
Patient eligibility criteria: The study inclusion and exclusion criteria are as follows
Inclusion Criteria: 1) Age ≥21 years; 2) Clinical diagnosis of T2DM and HbA1c ≥8% at the screening visit; 3) Self-identified as AA; 4) Subject must be taking an oral medication or insulin for diabetes to be able to assess medication adherence; 5) Subjects must be able to communicate in English; 6) Subjects must have access to a telephone (landline or cell phone) and/or ethernet for the study period; and 7) Subjects must be willing to use the FORA monitoring system for 3 months.
Exclusion Criteria: 1) Mental confusion on interview suggesting significant dementia; 2) Participation in other diabetes clinical trials; 3) Alcohol or drug abuse/dependency; 4) Active psychosis or acute mental disorder; and 5) Life expectancy <12 months.

Description of the Financial Incentive augmented Telephone-Delivered Diabetes Education and Skills Training Intervention: The financial incentives augmented telephone-delivered education and skills training intervention is comprised of two components: financial incentives and telephone-delivered education and skills training. Details of each component are provided below.
1. ***The Telephone-Delivered Diabetes Education and Skills Training Component***. Subjects will receive weekly telephone-delivered diabetes knowledge/information, patient activation, patient empowerment, and behavioral skills training delivered via telephone. The intervention will be delivered by telephone once a week for 12 weeks with each session lasting ~30 minutes.
2. ***The Financial Incentives Component***.
Structure of Incentives: The reward system is structured as follows:

- Low Frequency Financial Incentive – Group 1: the low frequency incentive structure will receive a reward for absolute percentage drops in HbA1c from baseline at 3-month follow-up, up to $300.
  - After 3 months, if their HbA1c has dropped 2% from baseline, or absolute HbA1c is 7%, they will receive a reward of $300, for a 1% drop, or an absolute HbA1c between 7 and 8 they will receive a reward of $150.

- Moderate Frequency Financial Incentive – Group 2: the moderate frequency incentive structure will receive a reward or uploading glucose measurements, and absolute percentage drops in HbA1c from baseline at 3-month follow-up, up to $300.
  - Each week participants in Group B can receive up to $10 for uploading glucose measurements and having good glucose control throughout the week. For each day they upload at least one glucose measurement, they will receive $1 (up to $7 at the end of the week). If they upload measurements every day of the week and their average glucose measurements at the end of the week are 150 or below they will receive an additional $3. Up to $10 per week for 3-months.
  - After 3 months, if their HbA1c has dropped 2% from baseline, or absolute HbA1c is 7%, they will receive a reward of $170, for a 1% drop, or an absolute HbA1c between 7 and 8 they will receive a reward of $85.

- High Frequency Financial Incentive – Group 3: the high frequency incentive structure will receive a reward or uploading glucose measurements, attending educational sessions, and absolute percentage drops in HbA1c from baseline at 3-month follow-up, up to $300.
  - Each week participants in Group A can receive up to $10 for uploading glucose measurements and having good glucose control throughout the week. For each day they upload at least one glucose measurement, they will receive $1 (up to $7 at the end of the week). If they upload measurements every day of the week and their average glucose measurements at the end of the week are 150 or below they will receive an additional $3. Up to $10 per week for 3-months.
  - Participants can also earn $5 each week if they attend the educational session. Educational sessions will last for 8 weeks, so they can receive up to $5 per week for 8 weeks.
  - After 3 months, if their HbA1c has dropped 2% from baseline, or absolute HbA1c is 7%, they will receive a reward of $130, for a 1% drop, or an absolute HbA1c between 7 and 8 they will

receive a reward of $65.

Thus, the maximum reward for each subject will be $300 over the 3 months of intervention and follow up.

Data Collection Strategy

Personnel: Two half-time masters-level trained diabetes educators (DEs) will deliver the interventions; one full-time research assistant (RA) will conduct screening, consent, enrollment procedures, and questionnaire administration; a half-time data entry clerk (DC) will be responsible for data entry; and a part-time masters-level statistician will be responsible for data management (DM). Each potentially eligible patient will be asked to report for a screening visit. At this visit, the following will be collected: basic demographic information, screening information, and a blood sample for a screening hemoglobin A1c (HbA1c) value. Basic demographic information will be collected to compare those screened to those randomized. Individuals who have a screening HbA1c of ≥8 will be asked to return within two weeks for a baseline visit. At the baseline visit, a full battery of questionnaires will be administered, and individuals will be randomized. All individuals randomized will have follow-up visits at 3 months, where survey questionnaires will be administered, and blood collected for HbA1c. Blood pressure will also be measured at the baseline and 3 months follow-up visit.

Primary Outcome Measure: ***HbA1c***: About 10cc of blood will be drawn by trained phlebotomists and sent to the lab for HbA1c.

Secondary Outcome Measures: ***Blood Pressure:*** Blood pressure readings will be obtained using automated BP monitors (OMRON IntelliSense™ HEM-907XL). The device will be programmed to take 3 readings at 2 minute intervals, and give an average of the 3 BP readings. ***Resource Utilization & Cost:*** Previously validated questions on resource utilization will be administered. The questionnaires are 1 page long and capture information on hospitalizations, physician/professional visits, and medications. ***Quality of Life:*** The SF-12 (Ware 1996) is a valid and reliable instrument to measure functional status. The SF-12 items reproduce at least 90% of the variance in PCS-36 and MCS-36 scores.

Process/Behavioral Measures: ***Information:*** This will be measured by the 24-item Diabetes Knowledge Questionnaire (DKQ) (Garcia et al, 2001), which has reliability coefficient of 0.78, showed sensitivity to a diabetes knowledge intervention, and has been tested in an ethnic minority group. ***Motivation:*** This will be measured by the 8-item Diabetes Empowerment Scale-Short Form (DES-SF), which has a Cronbach's alpha of 0.85 (Anderson et al., 2003). ***Self-Efficacy:*** This will be measured by the 8-item perceived diabetes self-management scale (PDSMS) (Wallston et al., 2007), which has Cronbach's alpha of 0.83 indicating internal consistency. ***Behavioral Skills:*** This will be assessed with the Summary of Diabetes Self-Care Activities (SDSCA) scale (Toobert 2000), a brief, validated self-report questionnaire of diabetes self-care. ***Treatment Credibility:*** This will be assessed using a modified treatment credibility scale developed by Borkovec and Nau, but modified for diabetes intervention research (Egede 2010). ***Delay Discounting:*** This will be measured using the 10-item self-report Quick Delay Questionnaire (QDQ) developed to test two factors: 1) delay discounting and 2) delay aversion. Responses and attitudes toward delay activities and situations relevant to every day adult life are assessed. Individual respond on a five-point Likert scale with a score of one representing, *"very like me"*, and five representing, *"not like me at all"*. Test-retest for the two factors are $r = 0.80$ for delay discounting and $r = 0.81$ for delay aversion. Internal consistency is satisfactory for both factors (delay discounting—$\alpha = 0.68$; delay aversion—$\alpha = 0.77$) (Clare et al., 2010).

Covariates: ***Demographics:*** Previously validated items from the National Health Interview Survey will be used to capture demographic characteristics. ***Social support:*** The Medical Outcomes Study (MOS) Social Support Survey (Sherbourne & Stewart 1991) will be used to measure social support. The total scale (α=0.97) has high internal consistency, good criterion and discriminant validity, and one-year test-retest reliability (0.72 to 0.76). ***Health Literacy:*** This will be assessed by the abbreviated version of the Test of Functional Health Literacy in Adults (S-TOFHLA) (Baker et al, 1999). The S-TOFHLA has a 0.80 correlation with the Rapid Estimate of Adult Literacy in Medicine (REALM). ***Depression:*** The PHQ-9 is a brief questionnaire that scores each of the 9 DSM-IV criteria for depression as "0" (not at all) to "3" (nearly every day). PHQ-9 score > or =10 have a sensitivity of 88% and a specificity of 88% for major depression (Kroenke 2001). ***Medical Comorbidity:*** The

patient's history of medical comorbidity will be documented using Chronic Health Conditions previously validated items from the Behavioral Risk Factor Surveillance System (BRFSS, 2010). ***Diabetes Fatalism:*** This will be measured with the 12-item Diabetes Fatalism Scales (DFS) (Egede 2009), a valid and reliable measure of diabetes fatalism (α=.804) that is associated with self-care problems, poor glycemic control, and decreased quality of life.

Patient Randomization: A permuted block randomization method will be used to assign subjects to one of the three intervention groups 1) High Frequency, 2) Moderate Frequency, and 3) Low Frequency. Block size will be varied to minimize the likelihood that the blind will be broken. The randomization will be stratified by baseline HbA1c levels (8-10% vs. >10%). Using REDCap, RAs will collect eligibility information and enter the information into the study database via the secured study website. Once eligibility is confirmed, the computer will generate the intervention assignment based on the pre-programmed randomization scheme. All subjects who are randomized will be entered into the study database and analyzed according to CONSORT guidelines (Altman 2001).

Sample Size and Power. For the primary/secondary efficacy outcomes, HbA1c (Primary) and BP/QOL (Secondary), with 18 subjects randomized to each of the three intervention groups, we will have 85% power to detect at least a 1.0 standardized effect size (difference in within group means in sd units). This calculation assumes that the primary/secondary efficacy outcomes are measured at 2 time points (baseline and 3-months); the intra-class correlation for repeated observations is no greater than 0.5; level of significance (α) =0.05, two-tailed test. Assuming that HbA1c measurements have a standard deviation of approximately 1.5 based on our pilot studies, the study has 85% power to detect a difference of 1.0 percentage points (raw units) change in HbA1c within each of the three comparisons groups. Analyses for continuous secondary/exploratory outcomes (behavioral and process variables) also have 85% power to detect a standardized effect size of 1.0sd. To account for the “fraction of missing” information that must be imputed in the ITT sample and the dilution effect of ITT analyses (Hsieh et al., 2003) the sample size is inflated by 10% to achieve a final ITT sample size of <u>20 subjects randomized to each treatment group (N=60)</u>.

Data Analysis
Design/Analysis Issues
<u>Analysis sets.</u> The intent-to-treat (ITT) sample, comprising all randomized patients, will be used for the primary analyses. The per protocol/completer sample will comprise subjects who were compliant with protocol requirements and for whom all required measurements over 3-months of follow-up have been made. Analyses will be carried out separately for ITT and per protocol samples to test sensitivity of conclusions to drop-outs/nonadherence. If differences are present between the per protocol and ITT analysis sets, the characteristics of the two analysis populations will be examined to aid in explaining any discrepancies.
Analysis Plan for Primary Aim (Efficacy): To test the efficacy of three financial incentive structures in combination with technology intensified diabetes education and skills training intervention on HbA1c levels in AAs with T2DM. Preliminary analyses will be conducted to assess the similarity of the treatment arms based on collected variables. Univariate descriptive statistics and frequency distributions will be calculated, as appropriate for all variables along with two-sided 95% confidence intervals when appropriate. Study discontinuation (attrition) rates will be estimated for each group. The preliminary analyses will describe the pre-specified covariates as well as identify potential confounding (prognostic) variables to be used as covariates in subsequent secondary analyses. Covariates to be examined in the preliminary analyses and potentially used in secondary analyses include age, sex, income, health literacy, social support, depression, and comorbidity. The primary framework for analysis is paired t-test. Change in HbA1c from baseline to 3-months (dependent variable) will be examined in each treatment arm (high, moderate and low frequency of financial incentives) using a type I error rate of 0.05.

Analysis Plan for Secondary Aims:
1. To test the efficacy of three financial incentive structures in combination with technology intensified diabetes education and skills training intervention on blood pressure and quality of life in AAs with T2DM.

2. To test the efficacy of three financial incentive structures in combination with technology intensified diabetes education and skills training intervention on diabetes knowledge and self-care behaviors (medication adherence, diet, exercise).

Similar to the primary analysis, paired t-test will be used to evaluate change in BP, QOL, knowledge and self-care variables from baseline to 3-months (dependent variables) within each treatment arm (high, moderate and low frequency of financial incentives) using a type I error rate of 0.05.

Analysis Plan for Exploratory Analyses. Exploratory analyses to examine frequency of session attendance, frequency of uploads and average weekly glucose within the three different financial incentive structures. Univariate descriptive statistics and frequency distributions will be calculated, as appropriate for all variables along with two-sided 95% confidence intervals when appropriate.

HUMAN SUBJECTS RESEARCH

PROTECTION OF HUMAN SUBJECTS

<u>1. RISKS TO THE SUBJECTS</u>

a. <u>Human Subjects Involvement and Characteristics</u>

The overarching aim of this proposal is test the efficacy of three financial incentive structures in combination with technology intensified diabetes education and skills training intervention on blood pressure and quality of life in AAs with T2DM. 60 AAs with T2DM will be randomized to three groups with varying frequency of financial incentives: 1) High Frequency: financial incentives for weekly uploads plus average glucose, incentives for weekly attendance to educational sessions, and incentives at the end of the study for meeting HbA1c goals 2) Moderate Frequency: financial incentives for weekly uploads plus average glucose, and incentives at the end of the study for meeting HbA1c goals, and 3) Low Frequency: financial incentives at the end of the study for meeting HbA1c goals.

<u>Study clinics</u>: The study sites for this study are MUSC general medicine, endocrine, family medicine, and community primary care clinics.
<u>Patient eligibility criteria</u>: The study inclusion and exclusion criteria are as follows
<u>Inclusion Criteria</u>: 1) Age ≥21 years; 2) Clinical diagnosis of T2DM and HbA1c ≥8% at the screening visit; 3) Self-identified as AA; 4) Subject must be taking at least one oral medication or insulin for diabetes to assess medication adherence; 5) Subjects must be able to communicate in English; 6) Subjects must have access to a telephone (landline or cell phone) and/or Ethernet for the study period; and 7) Subjects must be willing to use the FORA monitoring system for 3 months.
<u>Exclusion Criteria</u>: 1) Mental confusion on interview suggesting significant dementia; 2) Participation in other diabetes clinical trials; 3) Alcohol or drug abuse/dependency; 4) Active psychosis or acute mental disorder; and 5) Life expectancy <12 months.

b. <u>Sources of Materials</u>

1. Research Material & Data: Sources of research material include medical history, research questionnaires, blood pressure readings, and blood specimens. The questionnaires will obtain information about demographics, clinical history, diabetes self-care, resource use, depression, and quality of life. Patients will provide ~10cc of blood for laboratory testing.
2. Linkages to Subjects**:** Subjects will provide identifying information in addition to research data. Paper documents pertaining to this study will be stored in locked file cabinets in both the clinical center and the data management center, and data will be entered into secure, password-protected web databases developed for this study. A database of name, contact address, telephone number, and other research identification numbers will be stored separate from the study database, for purposes of audit by the sponsor (NIH) and MUSC IRB, if necessary. Access to study data will be limited to research personnel.
3. Collection of Data and Specimens:

Personnel: Two half-time masters-level trained diabetes educators (DEs) will deliver the interventions; one full-time research assistant (RA) will conduct screening, consent, enrollment procedures, and questionnaire administration; a half-time data entry clerk (DC) will be responsible for data entry; and a part-time masters-level statistician will be responsible for data management (DM).

Potential Risks

Potential risks to the patient include possible violation of the patient's privacy, discomfort with questions on the research questionnaire, discomfort and bleeding from blood draws, discomfort with BP measurement, and psychological distress. Details on how these risks will be minimized are discussed under adequacy of protection against risks below.

ADEQUACY OF PROTECTION AGAINST RISKS

a. Recruitment and Informed Consent

We will use two complementary approaches to identify eligible study subjects. The first method will consist of systematic identification of AA patients with T2DM. After obtaining IRB approval for a partial waiver of HIPAA, we will use clinic-billing records over the previous 12-month period to identify AA subjects with ICD-9 codes consistent with a diagnosis of type 2 diabetes. The physicians of eligible patients will be notified of their patients' potential eligibility and asked permission to enroll their patients in this study. After consent is obtained from the physicians, letters of invitation on clinic letterhead signed by the patient's physician will be mailed to patients from the study clinics. The letter will provide information about the study, explain the study requirements, and clarify that only subjects that meet certain criteria will be eligible to participate in the study. The letter will include an addressed and stamped post-card that subjects can mail back to indicate interest or lack of interest in participating in the study. In addition, the letter will provide a telephone number that interested subjects can call to receive detailed information about the study. In the letter, subjects will also be informed that they will receive a follow-up call in two weeks unless they mail back the post card or call to decline being contacted. Subjects that mail back the post card and express interest or call the provided telephone number will receive detailed information about the study. Subjects who agree to participate will be asked to provide written consent and will be scheduled for the initial screening assessment. The second method will consist of referrals from physicians, other clinic staff such as nurses, or patients themselves in response to recruitment flyers for the study. The PI will share the goals of the study and inclusion/exclusion criteria with physicians and clinic staff during clinic administrative meetings. Physicians and clinic staff will be asked to refer appropriate subjects to the study research assistants. In addition, IRB approved recruitment flyers will be posted in prominent locations in the study clinics.

Protection against Risk

A. Patients will be protected against potential risks as follows:

1. Psychological Distress: Because we will be administering a questionnaire that measures the presence of depression, we will take several steps to ensure the safety of all research participants. RAs will be trained by the PI to identify patients who meet criteria for depression on the PHQ-9. Subjects' who screen positive for depression will be notified during the visit and verbally instructed to seek care from their primary health care provider (PCP). They will also be given the 24-hour MUSC crisis psychiatry telephone service and told to call if they experience acute worsening of symptoms before they can be seen by their PCP.

2. Venipuncture (blood drawing): To reduce the risks of discomfort and bruising, venipuncture will be performed by trained personnel. To reduce the risk of fainting, blood will be drawn while subjects are in a seated position. The amount of blood that will be drawn, approximately 10cc, is not considered to pose a health risk for most adults. .

3. Blood Pressure Measurement: To lessen any associated risks, blood pressure measurements will be performed only by trained personnel utilizing a standardized protocol. Subjects with elevated blood pressure will be advised to contact their primary care provider. Those with potentially life threatening blood pressure readings will be sent to a local emergency room for treatment.

4. Administration of Research Questionnaires: Some participants might be offended by detailed questions about emotional or physical health status and impairment, and healthcare utilization. All participants will be informed at the outset that they may terminate participation at any point. Our past research suggests that data

collection using these measures can be conducted without undue psychological distress or exacerbation of symptoms among study participants.
5. Unknown risks: Subject participation in research may have other unknown risks. The researchers will advise subjects if they learn of emerging information that might alter subjects' decisions to participate in this study.

B. Subjects requiring medical or other professional intervention for study-related events will be provided with appropriate and timely medical guidance by the designated medical monitor (Dr. Ozieh – board certified internist). Dr. Ozieh will have oversight on medical risks and will review all adverse events and report them to the IRB in accordance with the MUSC IRB Adverse Event Reporting Policy. The results of subjects' clinical assessments will be available within a few days of their study visit. Dr. Ozieh will review and advise subjects of these results by phone and, at their request; will also advise their personal physician of the results.

C. To protect against the potential risk of loss of confidentiality and/or breach of privacy, data will be compiled using codes in lieu of personal identifiers. Access to study data will be limited to research personnel. Development of and security oversight for the electronic database for this study will be performed by the study statistician. Paper documents pertaining to this study will be stored in locked file cabinets and electronic data will be entered into secure, password-protected databases developed for this study by the research assistants. The PI will perform periodic review of the data entry process to ensure accuracy of recording. When study results are published or presented, only aggregate reports of the results will be used and participants' identity will not be revealed. A file of name, contact address, telephone number, and other research identification numbers will be stored separately on paper and on computer, for purposes of audit by the sponsor (NIH) and MUSC IRB, if necessary.

3. POTENTIAL BENEFITS OF THE PROPOSED RESEARCH TO THE SUBJECTS AND OTHERS

The intervention is expected to benefit patients, by increasing their knowledge of diabetes, activating and empowering them to better care for their diabetes, improving blood glucose and blood pressure control, and reducing their risk of developing complications of diabetes. Patients in the usual care group will benefit by increasing their general health knowledge.

4. IMPORTANCE OF KNOWLEDGE TO BE GAINED

The proposed study is innovative for a variety of reasons. First, behavioral economics is an emerging area of research that is not well tested in diabetes. Even though it is generally accepted that financial incentives can act as motivators to effect change in personal health behaviors (Sutherland 2008), few studies have assessed the effect of financial incentives on improving metabolic outcomes in T2DM. More importantly, there is little data on the effectiveness of financial incentives as motivators of behavior change in AAs. Second, the culturally-tailored education and skills training intervention proposed in this study is innovative because it targets patient level factors for which there is strong evidence of differences between AAs and Whites with T2DM (i.e. diabetes knowledge, self-management skills, empowerment, and fatalism/perceived control) (Egede 2005, Egede 2004, Nwasuruba 2007, Anderson 1995, Egede 2010). In addition, the intervention focuses on four key behaviors (physical activity, diet, medication adherence, and self-monitoring of blood glucose); maintains adequate dose and intensity of the intervention by delivering the intervention weekly for 12 weeks. Third, there are no large RCTs that have examined whether financial incentives augmented telephone-delivered diabetes education and skills training intervention will lead to greater improvements in metabolic control compared to usual care. Finally, the exploratory analyses will enable us to examine the effect of financial incentives augmented education and skills intervention on self-care behaviors and the mediation analysis will allow us to examine the contribution of self-care behaviors to improvements in metabolic control in African Americans with poorly controlled type 2 diabetes. With this approach, we can explore further targets for intervention in high risk African Americans with poorly controlled diabetes.

5. DATA AND SAFETY MONITORING PLAN

The data and safety monitoring plan will include an internal Data Safety Monitoring Committee (DSMC) and the institutional IRB. The purpose of the DSMC and IRB are to ensure the safety of participants and the validity and integrity of the data. Summaries of adverse events or patient safety concerns raised by the DSMC or IRB

will be made to NIH in the yearly progress unless the nature of a particular event is such that it bears reporting to NIH immediately.

DSMC: The internal DSMC will consist of the PI, biostatistician, co-investigators/consultants on the proposal, and a designated medical monitor (Dr. Ozieh – board certified internist who will have oversight on medical risks and review adverse events). The functions of the DSMC will include: 1) provide scientific oversight; 2) review all adverse effects or complications related to the study; 3) monitor accrual; 4) review summary reports relating to compliance with protocol requirements; and 5) provide advice on resource allocation. The DSMC will meet quarterly and as necessary by telephone. The recommendations of the DSMC will be reviewed and the PI will take appropriate corrective actions as needed.

Institutional IRB: The IRB will review and approve the funded protocol, review patient consent forms, ensure protection of patient privacy and safety, and monitor the study on an on going basis. Adverse events will be reported to the IRB as they occur. Annual reports to the IRB will indicate accrual rate, adverse events, new findings that may influence continuation of the study, and reports of the DSMC.

BIBLIOGRAPHY & REFERENCES CITED

Altman DG, Schulz KF, Moher D, et al. The revised CONSORT statement for reporting randomized trials: explanation and elaboration. *Ann Intern Med.* Apr 17 2001;134(8):663-694.

American Diabetes Association. Standards of medical care in diabetes--2011. *Diabetes Care.* Jan;34 Suppl 1:S11-61.

Anderson RM, Fitzgerald JT, Gruppen LD, Funnell MM, Oh MS. The Diabetes Empowerment Scale-Short Form (DES-SF). *Diabetes Care.* May 2003;26(5):1641-1642.

Anderson RM, Funnell MM, Butler PM, Arnold MS, Fitzgerald JT, Feste CC. Patient empowerment. Results of a randomized controlled trial. *Diabetes Care.* Jul 1995; 18(7):943-949.

Baker DW, Williams MV, Parker RM, Gazmararian JA, Nurss J. Development of a brief test to measure functional health literacy. *Patient Educ Couns.* Sep 1999;38(1):33-42.

Baron RM, Kenny DA. The moderator-mediator variable distinction in social psychological research: conceptual, strategic, and statistical considerations. *J Pers Soc Psychol.* Dec 1986; 51(6):1173-1182.

Behavioral Risk Factor Surveillance System (2010). Survey Questionnaire, Behavioral Risk Factor Surveillance System, 2010. Centers for Disease Control and Prevention. Available at: http://www.cdc.gov./brfss/questionnaires/questionnaires.htm.
Bellg AJ, Borrelli B, Resnick B, et al. Enhancing treatment fidelity in health behavior change studies: best practices and recommendations from the NIH Behavior Change Consortium. Health Psychol. Sep 2004;23(5):443-451.

Bellg AJ, Borrelli B, Resnick B, et al. Enhancing treatment fidelity in health behavior change studies: best practices and recommendations from the NIH Behavior Change Consortium. Health Psychol. Sep 2004;23(5):443-451.

Blair SN, Haskell WL, Ho P, et al. Assessment of habitual physical activity by a seven-day recall in a community survey and controlled experiments. Am J Epidemiol. Nov 1985; 122(5):794-804.

Block G, Hartman AM, Dresser CM, Carroll MD, et al. A data-based approach to diet questionnaire design and testing. American Journal of Epidemiology. 1986; 124:453-469.

Block G, DiSogra C. WIC Dietary Assessment Validation Study, Final Report 1995. Available from Food and Nutrition Service, U.S. Department of Agriculture, 3101 Park Center Drive, Room 607, Alexandria, VA 22302.

Block G, Thompson FE, Hartman AM, Larkin FA, Guire KE. Comparison of two dietary questionnaires validated against multiple dietary records collected during a 1-year period. J Am Diet Assoc 1992; 92:686-693.

Block G, Woods M, Potosky A, Clifford C. Validation of a self-administered diet history questionnaire using multiple diet records. J Clin Epidemiol 1990; 43:1327-1335.

Borrelli B, Sepinwall D, Ernst D, et al. A new tool to assess treatment fidelity and evaluation of treatment fidelity across 10 years of health behavior research. J Consult Clin Psychol. Oct 2005;73(5):852-860.
Byar D. and Piantidosi S. Factorial Designs for Randomized Clinical Trials. Cancer Treatment Reports 1985; 69(10): 1055-1063.

CDC Diabetes Cost-Effectiveness Group. 2002. Cost-effectiveness of Intensive Glycemic Control, Intensified Hypertension Control, and Serum Cholesterol Level Reduction for Type 2 Diabetes. JAMA 287(19):2542-2551.

Clare S, Helps S, & Sonuga-Barke EJS. The quick delay questionnaire: a measure of delay aversion and discounting in adults. *ADHD Atten Def Hyp Disord* 2010;2:43-48.

Critchfield TS, Kollins SH. Temporal discounting: basic research and the analysis of socially important behavior. *J Appl Behav Anal.* Spring 2001;34(1):101-122.

Dall TM, Zhang Y, Chen YJ, Quick WW, Yang WG, Fogli J. 2010. The Economic Burden of Diabetes. Health Affairs 29(2): 1-7.

Egede LE, Strom JL, Durkalski VL, Mauldin PD, Moran WP. Rationale and design: telephone-delivered behavioral skills interventions for Blacks with Type 2 diabetes. 2010, *Trials.*11:35.

Egede LE, Dagogo-Jack S. Epidemiology of type 2 diabetes: focus on ethnic minorities. *Med Clin North Am.* Sep 2005; 89(5):949-975, viii.

Egede LE, Poston ME. Racial/ethnic differences in leisure-time physical activity levels among individuals with diabetes. *Diabetes Care.* Oct 2004; 27(10): 2493-2494.

Egede LE, Mueller M, Echols CL, Gebregziabher M. Longitudinal differences in glycemic control by race/ethnicity among veterans with type 2 diabetes. *Med Care.* Jun 2010;48(6):527-533.

Egede LE, Ellis C. Development and psychometric properties of the 12-item diabetes fatalism scale. *J Gen Intern Med.* Jan;25(1):61-66.

Fisher JD, Fisher WA: The information-motivation behavioral skills model. In: *Emerging theories in health promotion practice and research: strategies for improving public health* Edited by RJ D, RA C, MC K. Indianapolis, IN: Jossey-Bass, Inc; 2002.

Fisher JD, Fisher WA: Theoretical approaches to individual-level change. In: *Handbook of HIV Prevention.* Edited by Peterson JL, DiClemente RJ. New York: Kluwer Academic/Plenum Press; 2000: 3-55.

Fisher WA, Fisher JD, Harman J: The Information-Motivation-Behavioral Skills Model: A general social psychological approach to understanding and promoting health behavior. In: *Social Psychological Foundations of Health and Illness.* Edited by Suls J, Wallston KA. Malden, MA: Blackwell; 2003: 82-106.

Funnell MM, Brown TL, Childs BP, et al. National standards for diabetes self-management education. *Diabetes Care.* Jan;33 2010, Suppl 1:S89-96.

Garcia AA, Villagomez ET, Brown SA, Kouzekanani K, Hanis CL. The Starr County Diabetes Education Study: development of the Spanish-language diabetes knowledge questionnaire. *Diabetes Care.* Jan 2001;24(1):16-21.

Gold MR, Siegel JE, Russell, LB, Weinstein MC. (eds). 1996. Cost-effectiveness in Health and Medicine. New York: Oxford University Press.

Green L, Myerson J. A discounting framework for choice with delayed and probabilistic rewards. *Psychol Bull.* Sep 2004;130(5):769-792.

Hedeker D, Gibbons, RD. *Longitudinal Data Analysis*. New York: John Wiley and Sons, Inc.; 2006.

Hsieh FY, Lavori PW, Cohen HJ, Feussner JR. An overview of variance inflation factors for sample-size calculation. Eval Health Prof. 2003 Sep;26(3):239-57.

Kane RL, Johnson PE, Town RJ, Butler M. A structured review of the effect of economic incentives on consumers' preventive behavior. *Am J Prev Med.* Nov 2004;27(4):327-352.

Kroenke K, Spitzer RL, Williams JB. The PHQ-9: validity of a brief depression severity measure. J Gen Intern Med. Sep 2001;16(9):606-613.

Krousel-Wood M, Islam T, Webber LS, Re RN, Morisky DE, Muntner P. New medication adherence scale versus pharmacy fill rates in seniors with hypertension. *Am J Manag Care.* Jan 2009;15(1):59-66.

Lichstein KL RB, Grieve, R. Fair tests of clinical trials: A treatment implementation model. *Advances in behavior research and therapy* 1994;16:1-29.

Little R, Rubin, D. *Statistical Analysis With Missing Data*. New York: John Wiley and Sons, Inc.; 1987.

National Institute of Diabetes and Digestive and Kidney Diseases. National Diabetes Statistics, 2007 fact sheet. Bethesda, MD: U.S. Department of Health and Human Services, National Institutes of Health, 2008.

Norris SL, Engelgau MM, Narayan KM. Effectiveness of self-management training in type 2 diabetes: a systematic review of randomized controlled trials. *Diabetes Care.* Mar 2001; 24(3):561-587.

Norris SL, Lau J, Smith SJ, Schmid CH, Engelgau MM. Self-management education for adults with type 2 diabetes: a meta-analysis of the effect on glycemic control. *Diabetes Care.* Jul 2002; 25(7):1159-1171.

Nwasuruba C, Khan M, Egede LE. Racial/ethnic differences in multiple self-care behaviors in adults with diabetes. *J Gen Intern Med.* Jan 2007;22(1):115-120.

Osborn CY, Rivet Amico K, Fisher WA, **Egede LE**, Fisher JD. An information-motivation-behavioral skills analysis of diet and exercise behavior in Puerto Ricans with diabetes. *J Health Psychol.* Nov 2010;15(8):1201-1213.

Osborn CY, **Egede LE**. Validation of an Information-Motivation-Behavioral Skills model of diabetes self-care (IMB-DSC). *Patient Educ Couns.* Apr 2009;79(1):49-54.

Osborn CY, Bains SS, **Egede LE**. Health literacy, diabetes self-care, and glycemic control in adults with type 2 diabetes. *Diabetes Technol Ther.* Nov 2010;12(11):913-919.

Pauly MV. Health Benefits at Work: An Economic and Political Analysis of Employment-based Health Insurance. Ann Arbor: University of Michigan Press; 2002.

Pauly MV, Held PJ. Benign moral hazard and the cost-effectiveness analysis of insurance coverage. J Health Econ 1990;9:447-61.

Piette JD, Weinberger M, Kraemer FB, McPhee SJ. Impact of automated calls with nurse follow-up on diabetes treatment outcomes in a Department of Veterans Affairs Health Care System: a randomized controlled trial. *Diabetes Care.* Feb 2001;24(2):202-208.

Preacher K, Hayes A. SPSS and SAS procedures for estimating indirect effects in simple mediation models. Behavior Research Methods, Instruments and Computers. 36(4), 717-731, 2004.

Rachlin H. The science of self-control. Cambridge: Harvard University Press, 2000.

Reynolds B. A review of delay-discounting research with humans: relations to drug use and gambling. *Behav Pharmacol.* Dec 2006;17(8):651-667.

Sallis JF, Haskell WL, Wood PD, et al. Physical activity assessment methodology in the Five-City Project. Am J Epidemiol. Jan 1985; 121(1):91-106.

Sarkar U, Piette JD, Gonzales R, et al. Preferences for self-management support: findings from a survey of diabetes patients in safety-net health systems. *Patient Educ Couns.* Jan 2008;70(1):102-110.

Shea S, Weinstock RS, Starren J, et al. A randomized trial comparing telemedicine case management with usual care in older, ethnically diverse, medically underserved patients with diabetes mellitus. *J Am Med Inform Assoc.* Jan-Feb 2006;13(1):40-51.

Sherbourne, C. D., & Stewart, A. L. (1991). The MOS Social Support Survey. Social Science and Medicine, 32, 705-14.

Schoenbaum M, Unutzer J, Sherbourne C, Duan N, Rubenstein LV, Miranda J, Meredith LS, Carney MF, Wells K. 2001. Cost-effectiveness of practice-initiated quality improvement for depression: results of a randomized control trial. JAMA. 286(11):1325-1330.

Sutherland K, Christianson JB, Leatherman S. Impact of targeted financial incentives on personal health behavior: a review of the literature. *Med Care Res Rev.* Dec 2008;65(6 Suppl):36S-78S.

Toobert DJ, Hampson SE, Glasgow RE. The summary of diabetes self-care activities measure: results from 7 studies and a revised scale. Diabetes Care. Jul 2000; 23(7):943-950.

U.S. Department of Veterans Affairs. Estimating VA Treatment Cost: Methods and Applications, special Supplement to the September 2003 issue of the journal Medical Care Research. Available online: http://www.herc.research.va.gov/publications/supplement_mcrr_2003.asp

Wallston KA, Rothman RL, Cherrington A. Psychometric properties of the Perceived Diabetes Self-Management Scale (PDSMS). *J Behav Med.* Oct 2007;30(5):395-401.

Ware JE, Kosinski M, and Keller SD. A 12-Item Short-Form Health Survey: Construction of scales and preliminary tests of reliability and validity. Medical Care, 1996; 34(3):220-233.

Weinberger M, Kirkman MS, Samsa GP, et al. A nurse-coordinated intervention for primary care patients with non-insulin-dependent diabetes mellitus: impact on glycemic control and health-related quality of life. *J Gen Intern Med.* Feb 1995;10(2):59-66.

Young RJ, Taylor J, Friede T, et al. Pro-active call center treatment support (PACCTS) to improve glucose control in type 2 diabetes: a randomized controlled trial. *Diabetes Care.* Feb 2005;28(2):278-282.
